# Clinical and genetic data of 22 new patients with *SMAD3* pathogenic variants and review of the literature

**DOI:** 10.1002/mgg3.1132

**Published:** 2020-03-10

**Authors:** Bertrand Chesneau, Thomas Edouard, Yves Dulac, Hélène Colineaux, Maud Langeois, Nadine Hanna, Catherine Boileau, Pauline Arnaud, Nicolas Chassaing, Sophie Julia, Guillaume Jondeau, Aurélie Plancke, Philippe Khau Van Kien, Julie Plaisancié

**Affiliations:** ^1^ Service de génétique médicale Hôpital Purpan CHU de Toulouse Toulouse France; ^2^ Centre de Référence du syndrome de Marfan et des syndromes apparentés Hôpital des Enfants CHU de Toulouse Toulouse France; ^3^ Département d'épidémiologie, d'économie de la santé et de santé publique CHU de Toulouse Toulouse France; ^4^ LEASP UMR1027 INSERM Université Toulouse III Toulouse France; ^5^ Centre de Référence pour le syndrome de Marfan et apparentés AP‐HP Hôpital Bichat Faculté Paris Diderot LVTS INSERM U1148 Paris France; ^6^ UF de Génétique Médicale et Cytogénétique Centre Hospitalier Régional Universitaire de Nîmes Nîmes France

**Keywords:** Aneurysms‐Osteoarthritis syndrome, Loeys–Dietz syndrome, *SMAD3*, TGFβ

## Abstract

**Background:**

Pathogenic *SMAD3* variants are responsible for a cardiovascular phenotype, mainly thoracic aortic aneurysms and dissections. Precocious identification of the vascular risk such as aortic dilatation in mutated patients has a major impact in terms of management, particularly to avoid dissection and sudden death. These vascular damages are classically associated with premature osteoarthritis and skeletal abnormalities. However, variable expressivity and incomplete penetrance are common with *SMAD3* variants.

**Methods:**

To investigate the clinical variability observed within *SMAD3* patients, we reviewed the phenotypic and genetic data of 22 new patients from our Centre and of 133 patients reported in the literature. From this cohort of 155 mutated individuals, we first aimed to delineate an estimated frequency of the main clinical signs associated with *SMAD3* pathogenic variants and, then, to look for genotype‐phenotype correlations, mainly to see if the aortic phenotype (AP) could be predicted by the *SMAD3* variant type.

**Results:**

We showed, herein, the absence of correlation between the *SMAD3* variant type and the occurrence of an AP in patients.

**Conclusion:**

Therefore, this report brings additional data for the genotype‐phenotype correlations of *SMAD3* variants and the need to explore in more detail the effects of genetic modifiers that could influence the phenotype.

## Graphical abstract

Pathogenic *SMAD3* variants are responsible for a cardiovascular phenotype, mainly thoracic aortic aneurysms and dissections, with variable expressivity and incomplete penetrance. To investigate the clinical variability observed within *SMAD3* patients, we reviewed the data of 22 new patients from our Centre and of 133 patients reported in the literature. We first aimed to delineate an estimated frequency of the main clinical signs associated with *SMAD3* pathogenic variants and, then, to look for genotype‐phenotype correlations. We showed herein the absence of correlation between the *SMAD3* variant type and the occurrence of an aortic phenotype in patients. This report brings additional data for the genotype‐phenotype correlations of *SMAD3* variants and the need to explore in more detail the effects of genetic modifiers that could influence the phenotype.

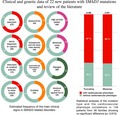

## 1 INTRODUCTION

Pathogenic *SMAD3* variants are responsible for a wide spectrum of clinical manifestations including Aneurysms‐Osteoarthritis Syndrome (Aubart et al., 2014). The *SMAD3* gene represents 5–10 percents of the transforming growth factor β (TGFβ) vasculopathies (TGFβvs), caused by a defect in the TGFβ pathway genes such as *TGFBR1*,* TGFBR2*,* TGFB2*,* TGFB3* and *SMAD2*. There is a clinical overlap between these TGFβvs and Marfan syndrome (MFS) in which the aortic aneurysm/dissection events dominate the cardiovascular phenotype. Unlike MFS, dilatation/dissection events are frequent in other arteries and arterial tortuosity is common, particularly in head and neck vessels (Aubart et al., 2014; Laar et al., 2012; Regalado et al., 2011). In addition, the main features that distinguish *SMAD3*‐related disorders (SRD) from MFS and other TGFβvs are precocious osteoarthritis (<50 years old) and Charcot–Marie–Tooth‐like neuropathy (Aubart et al., 2014).

The *SMAD3* gene contains two functional domains, Mad Homology 1 (MH1) and 2 (MH2), separated by a linker region (Schepers et al., 2018). The N‐terminal domain MH1 is mainly involved in DNA binding. The C‐terminal domain MH2 mediates oligomerization with SMAD4 and SMAD‐dependent downstream transcription (Schepers et al., 2018). Heterozygous variants of different nature (missense, truncating, splicing variants) have been described in SRD (Schepers et al., 2018). Interestingly, gain‐of‐function in overall TGFβ pathway has been observed in the aortic wall of patients with aortic aneurysms, including patients with loss‐of‐function mutations in the TGFβ pathway genes (Gomez et al., 2009). Despite the absence of hot spot identification in *SMAD3*, the majority (63) of the missense variants is reported in the MH2 domain.

Thus, the role of *SMAD3* in a wide phenotypic spectrum with different types of variants prompted us to review all the individuals with a *SMAD3* (likely) pathogenic variant from our Centre and to compare them with published cases, first to determine an estimated frequency of the main clinical signs in SRD and the presence of any genotype‐phenotype correlation associated with variants of this gene.

## 2 MATERIALS AND METHODS

### 2.1 Patients

All the 22 patients were referred to our Reference Centre for “Marfan syndrome and related disorders” for personal and/or family history suggestive of a connective tissue disorder (mainly aortic event or dilatation and skeletal findings).

This study was designed in compliance with the tenets of the Helsinki Declaration. Informed consent was obtained from all individuals included in this study.

We also assembled all the clinical data available on individuals with a pathogenic or likely pathogenic variant in the *SMAD3* gene from an exhaustive review of the literature using the keyword “SMAD3” in the PubMed database. Patients whose vascular phenotype was unknown (in particular, the presence or the absence of an aortic dilatation) were not included in the correlation study.

### 2.2 Molecular testing

Index cases benefited from multigene panels that include *SMAD3* (NM_005902.3) as well as a number of other genes associated with disorders that include heritable thoracic aortic aneurysms/dissections (Data S1). For the patient 9, for whom the *SMAD3* pathogenic variant was already known (family 4), a targeted Sanger sequencing was directly performed. Segregation analysis of any identified (likely) pathogenic variant was performed in each family (parents and other affected family members when available). All variants classified according to the ACMG recommendations (Richards et al., 2015) as pathogenic, likely pathogenic or of unknown significance (VUS) were reported here.

### 2.3 Phenotype classification

Phenotypes were classified into two groups: (a) an aortic phenotype (AP) that includes the aortic events (dissection, rupture, or elective repair of the aorta) or dilatation (more than two standard deviations measured by echocardiogram at the Valsalva sinus) and (b) a mild phenotype grouping asymptomatic patients and patients without aortic damage. Patients without AP but with an aneurysm in another artery were considered to have a mild phenotype.

### 2.4 Statistical analysis

From more than 300 patients reported in literature with SRD, only 133 of them were described with precise clinical data to be included in our genotype‐phenotype correlations study (Arno et al., 2012; Arroyave, Carretero, & Gruosso, 2018; Backer & Braverman, 2018; Campens et al., 2015; Kaadan et al., 2018; Laar et al., 2012; Nevidomskyte et al., 2017; Overwater et al., 2018; Proost et al., 2015; Regalado et al., 2011; Schepers et al., 2018; Wischmeijer et al., 2013; Ye et al., 2013). Six out of these 133 patients bearing splice variants in *SMAD3* (Campens et al., 2015; Nevidomskyte et al., 2017; Overwater et al., 2018) were not included in our analysis because the underlying pathogenic mechanism of these variants could not be predicted with certainty.

We studied the link between the variant type and the presence of an AP with logistic regression models. As some patients belonged to the same family, we also performed an analysis on the index cases only. *P* < .05 were considered as significant. Statistical analyses were performed with STATA release 14.

We also compared patients with a missense variant in either the MH1 or the MH2 domain to patients with a truncating variant. Four patients were not included in this analysis as they had a missense variant within the N‐terminal extremity before the MH1 domain.

## 3 RESULTS

### 3.1 Clinical and genetic data

**Figure 1:**
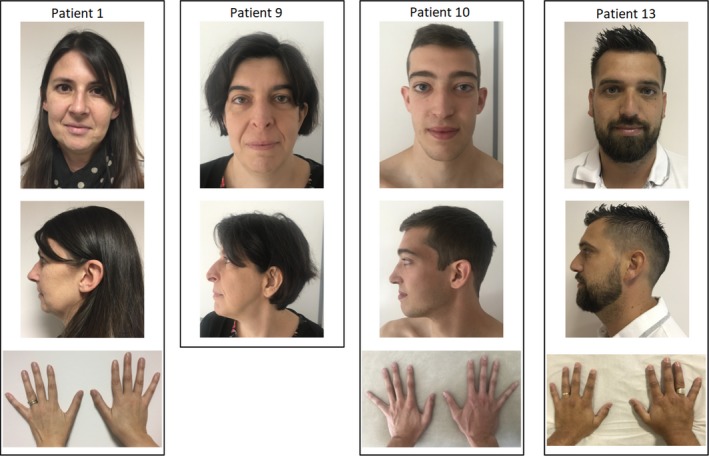
Photographs of patients 1, 9, 10 and 13, showing the high variability in craniofacial and extremities features

We identified 22 patients from eight families carrying a heterozygous (likely) pathogenic variant in *SMAD3* (Table 1; Figure 1; Data S2). Four were novel variants: c.269_271dup (p.Arg90dup) classified as pathogenic (PS2, PM1, PM2, PM4, PP1), c.736del (p.Glu246Arg*10) classified as likely pathogenic (PVS1, PM2), c.874del (p.Arg292Glufs*49) classified as pathogenic (PVS1, PM2, PP1) and c.991G>T (p.Val331Phe) classified as likely pathogenic (PM1, PM2, PM6, PP3).

**Table 1:** Genetic and clinical data in 22 patients with SRD and review of the literature

*SMAD3* variant	Family 1		Family 2			Family 3			Family 4			Family 5		Family 6	Family 7							Family 8		
	c.736del		c.269_271dup			c.1153A>G			c.334_335delinsCT			c.874del		c.788C>T	c.668delC							c.991G > T		
	p.Glu246Arg*10		p.Arg90dup			p.Arg385Gly			p.Ala112Leu			p.Arg292Glufs*49		p.Pro263Leu	p.Pro223Glnfs*18							p.Val331Phe		
Protein domain (exon)	MH2 (ex 6)		MH1 (ex 2)			MH2 (ex 8)			MH1 (ex 2)			MH2 (ex 7)		MH2 (ex 6)	MH2 (ex 6)							MH2 (ex 7)		
Patient	1	2	3	4	5	6	7	8	9	10	11	12	13	14	15	16	17	18	19	20	21	22	Our patients	Total (155)
Age (years)	40	6	39	10	8	60	38	32	48	19	17	52	32	7	59	19	58	22	18	33	32	20	28.8 (mean)	
Gender	F	M	F	M	M	M	M	M	F	M	F	F	M	M	M	M	M	F	F	M	M	M	*N* (%)	*N* (%)
Cardiovascular findings																								
Aortic aneurysm	−	−	+	−	+	+	+	−	−	+	−	+	+	+	+	−	+	−	−	−	−	+	11/22 (50)	104/155(67)
Aortic dissection, rupture	−	−	−	−	−	−	−	−	−	−	−	+	−	−	+	−	−	−	−	−	−	−	2/22 (9)	39/133 (29)
Aneurysm in cerebral art.	−	ND	−	ND	ND	ND	ND	−	−	ND	ND	+	ND	ND	−	ND	+	−	−	−	ND	−	2/11 (18)	16/83 (17)
Aneurysm in other vessels	−	ND	−	ND	ND	ND	ND	+	−	ND	ND	−	ND	ND	+	ND	−	−	−	−	ND	−	2/11 (18)	24/95 (25)
Arterial tortuosity	+	ND	−	ND	ND	ND	ND	ND	−	ND	ND	+	ND	ND	ND	ND	−	−	−	−	ND	−	2/9 (22)	19/45 (42)
Mitral valve prolapse	−	−	−	−	−	−	−	−	+	+	−	−	−	−	−	−	+	−	−	−	−	−	3/22 (14)	34/122 (28)
Aortic valve insufficiency	−	−	−	−	−	+	+	−	−	−	−	−	−	−	+	−	−	−	−	−	−	+	4/22 (18)	5/36 (14)
Skeletal findings																								
Dolichostenomelia	−	−	−	−	−	+	−	+	ND	+	−	ND	−	−	+	+	−	+	−	+	ND	+	8/19 (42)	15/72 (21)
Arachnodactyly	+	−	−	−	−	−	−	+	ND	+	+	ND	−	−	−	+	−	+	+	ND	ND	−	7/19 (37)	21/79 (27)
Scoliosis	−	−	−	−	−	+/−	+	−	−	+	+	+/−	−	−	+	+	+	−	+	+	−	+	9/22 (41)	41/95 (43)
Pectus deformity	−	−	−	−	−	−	+	+	−	−	−	ND	−	+	+	+	−	−	−	+	ND	−	6/20 (30)	24/93 (26)
Pes planus	−	−	ND	−	−	ND	+	ND	−	−	−	+	−	−	−	+	−	+	−	ND	ND	−	4/17 (24)	42/83 (51)
Joint laxity	+	−	−	−	−	−	+	−	+	−	−	+	−	+	−	+	+	+	+	ND		−	9/20 (45)	21/88 (24)
Osteoarthritis	+	−	ND	−	−	ND	−	ND	−	−	−	+	−	−	+	+/−	+	ND	−	ND	−	−	4/17 (24)	44/100 (44)
Osteopenia (*Z*‐score < −1) or osteoporosis (*Z*‐score < 2.5)	ND	ND	+(−1.3)	− (1.1)	− (0.2)	ND	ND	ND	ND	ND	− (−0.9)	ND	ND	− (−0.6)	ND	+ (−2.2)	+ (−2.0)	+ (−2.2)	+ (−1.5)	ND	ND	ND	5/9 (55)	7/16 (44)
Craniofacial																								
Facial features[Link]	−	−	−	−	−	−	−	+	−	+	+	ND	−	−	+	−	ND	−	ND	+	−	+	6/19 (32)	7/24 (29)
Hypertelorism	−	−	+/−	−	+	ND	−	ND	+	+	+	ND	−	−	−	ND	ND	−	ND	−	−	+	5/15 (33)	21/85 (25)
Abnormal uvula	+	−	−	−	−	−	−	−	ND	−	−	ND	−	−	−	+	−	−	−	−	ND	−	2/19 (11)	26/68 (39)
High arched palate	+	−	−	−	−	+	−	+	ND	+	+	ND	−	−	+	−	+	+	+	+	+	+	12/20 (60)	28/63 (44)
Skin																								
Velvety skin	+	−	+	−	−	−	+	+	ND	−	−	ND	+	+	−	−	+	+	+	+	ND	+	11/19 (58)	30/65 (46)
Striae	−	−	−	−	−	−	−	−	+	−	−	ND	−	−	−	+	−	+	−	+	ND	−	4/20 (20)	25/74 (34)
Ocular																								
Severe myopia	−	−	+	−	−	+	−	+	−	−	−	−	−	−	+	ND	−	−	−	−	−	−	4/21 (19)	6/39 (15)
Blue sclerae	−	−	−	−	−	−	−	−	ND	−	−	ND	−	−	−	+/−	−	−	−	−	−	−	0/20 (0)	0/22 (0)
Other																								
Pneumothorax	−	−	−	−	−	+	−	−	−	−	−	−	−	−	+	−	ND	−	−	ND	−	−	2/20 (10)	2/42 (5)
Allergy	+	−	+	−	ND	ND	−	ND	+	+	+	+	+	−	+	ND	+	−	+	ND	−	−	10/17 (58)	10/17 (58)
Ombilical/inguinal hernia	−	−	−	−	−	−	+	−	−	−	−	−	−	−	−	−	+	−	−	ND	−	+	3/21 (11)	34/100 (34)
Score of systemic features[Link]	3	0	1	0	0	4	4	7	2	7	5	ND	0	1	7	8	3	6	4	5	ND	4	4 (med)	

Abbreviations: +, present; −, absent; +/−, likely; art., arteries; ND, not determined; SRD, *SMAD3*‐related disorders.

According to the Revised Ghent Nosology for the Marfan syndrome (Loeys et al., 2010).

**Figure 2:**
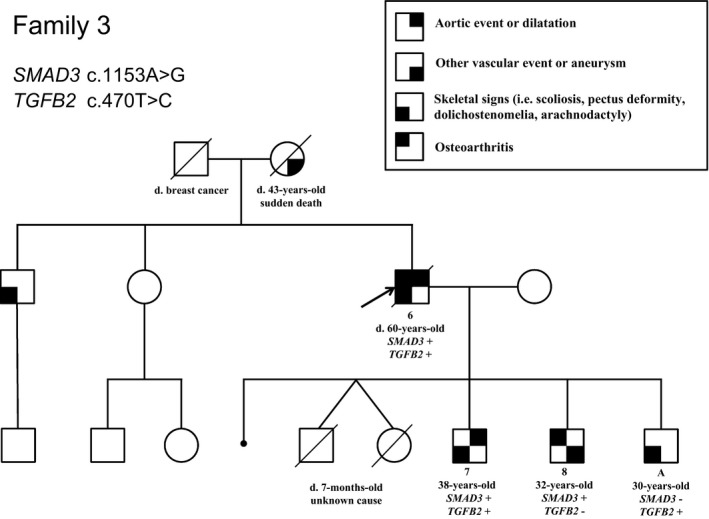
Pedigree of Family 3 with the segregation of the two heterozygous variants in *SMAD3* and *TGFB2* in patients 6, 7, 8 and A. The legend indicates the clinical features of the patients. The presence or the absence of the family variants in *SMAD3* and *TGFB2* is indicated by “+” or “−” after the gene name

Interestingly, in family 3, besides the c.1153A>G (p.Arg385Gly) variant identified in *SMAD3,* a second variant was identified in *TGFB2*: the c.470T>C (NM_001135599.2) (p.Ile157Thr) variant, which was classified as a VUS. Indeed, it has been reported at 0.0002481% in gnomAD and is predicted damaging by in silico analyses (Polyphen2, Mutation Taster) and tolerated by SIFT software. The index case in family 3 (patient 6) who was carrying both the heterozygous variants in *SMAD3* and *TGFB2*, displayed the most severe phenotype of the family as shown in the pedigree (Figure 2). He underwent surgery for an ascending aorta dilatation at 56 year old and suffered from aortic insufficiency, lumbar spondylolisthesis and scoliosis. He died at 60 years old from a pulmonary embolism. His elder son (patient 7) also displays a severe phenotype compared to his brothers, each carrying only the *SMAD3* (patient 8) or the *TGFB2* variant (patient A; Figure 2, Data S2). He has an aortic dilatation (41 mm, +3.7SD at the sinus of Valsalva), recurrent inguinal hernias and a severe maxillary hypoplasia. The patient 8 carrying only the *SMAD3* variant made a dissection of the left primitive iliac artery at 30 years old; he also had a dilatation in the right iliac artery. His echocardiography was normal. The echocardiography of the son carrying the *TGFB2* variant (patient A) did not reveal any aortic dilatation or valvular heart disease but hypertrophic cardiomyopathy with asymmetric thickening of the interventricular septum.

### 3.2 Genotype‐phenotype correlations

We compared 149 patients (127 + 22 patients) from 56 families (with 1–28 members) according to their aortic status and the nature of the *SMAD3* variant (truncating vs. missense variant; Data S3). After performing a statistical analysis, we did not detect any significant difference in the AP between patients carrying a truncating variant and patients with a missense variant in *SMAD3*. Both groups had indeed about 68% of patients with an aortic disease (*P* = .935). When comparing missense variants in the MH2 domain versus truncating variants, we also did not detect any statistical difference in the AP (*P* = .848). We did not observe a statistical difference either when comparing the phenotype of patients with a missense variant in the MH1 domain to those with a truncating variant (*P* = .656). We did not detect any significant difference either when analyzing the index cases only.

## 4 DISCUSSION

Pathogenic heterozygous variants in the *SMAD3* gene are a rare cause of connective tissue disorder. To our knowledge, 132 families have been reported in the literature with 102 different variants (Arno et al., 2012; Arroyave et al., 2018; Aubart et al., 2014; Backer & Braverman, 2018; Campens et al., 2015; Collins, Flor, Tang, Bange, & Zarate, 2018; Hostetler et al., 2019; Kaadan et al., 2018; Kfoury, Chen, & Lin, 2017; Laar et al., 2012; Nevidomskyte et al., 2017; Overwater et al., 2018; Proost et al., 2015; Regalado et al., 2011; Schepers et al., 2018; Wischmeijer et al., 2013; Ye et al., 2013) and four total or partial deletions of *SMAD3* (Hostetler et al., 2019; Schepers et al., 2018). In this work, we report 22 additional patients from 8 families, showing high variability in terms of expression and penetrance of *SMAD3* pathogenic variants even at pediatric age (Hostetler et al., 2019; Laar et al., 2012; Wischmeijer et al., 2013).

As shown previously (Aubart et al., 2014; Hostetler et al., 2019; Laar et al., 2012), aortic aneurysm and dissection are the main vascular findings, affecting, respectively 67% (104/155) and 29% (39/133) of all patients described with a *SMAD3* variant (Table 1). In our cohort, six out of the eight index cases have aortic aneurysms and 5 out of their 14 affected relatives display arterial aneurysms. Aortic dilatation can be found at a very young age (Arroyave et al., 2018; Aubart et al., 2014; Hostetler et al., 2019; Laar et al., 2012) as it is the case for patients 5 and 14 reported here (aged 8 and 7 years old, respectively) and patient 22 who underwent a surgery for an aortic dilation at 10 years old. Only 9% of our patients had an aortic dissection, the young age of some of our patients (5/22 are under 18) could be an explanation given that the aortic dissection has still not been reported in children to date. Nevertheless, one patient from a family with *SRD* died from an aortic dissection at the age of 18 (Wischmeijer et al., 2013). In absence of enough patients to correlate the risk of dissection with the phenotype and the gender, as it was done for *TGBFR1* and *TGFBR2* (Jondeau et al., 2016), the follow‐up and treatment of aortic manifestations in *SRD* is the same as in MFS. A recent study, reporting a cohort of 251 patients with *SMAD3* pathogenic variants found that the AP was less severe in *SMAD3* than in *TGFBR1* and *TGFBR2*, with later onset of aortic events (Hostetler et al., 2019). Aneurysms and dissections can also be seen in other arteries particularly intracranial aneurysms found in almost 20% of patients (16/83, Table 1). It is important to note that arterial events can happen even in the absence of aortic involvement: 14 patients of our study displayed an extra‐aortic aneurysm or dissection history without aortic anomalies (i.e. patient 8 who made an iliac artery dissection with a normal aortic diameter). Therefore, ultrasonography of supra‐aortic vessels is also recommended as well as brain and abdominopelvic magnetic resonance angiography.

One of our patients (patient 22, Data S2) presents with a congenital heart malformation. Other patients have been reported with various congenital heart malformations including ventricular septal defect and hypoplastic left heart syndrome but these malformations remain rare in *SRD* (Fitzgerald, Bhat, Conard, Hyland, & Pizarro, 2014; Hostetler et al., 2019; Laar et al., 2012; Overwater et al., 2018). Four of our patients display aortic insufficiency. To our knowledge, aortic insufficiency has been reported only once in the literature (Arroyave et al., 2018).

Premature osteoarthritis is very frequent in *SRD* with almost half of the mutated patients suffered from osteoarthritis (44/100, Table 1). Interestingly, *SMAD3* polymorphisms have recently been linked to the osteoarthritis risk (Hong et al., 2018). The key role of *SMAD3* and the TGFβ pathway in maintaining joint cartilage and preventing osteoarthritis has been reported several times (Chen, Thuillier, Chin, & Alliston, 2012), especially in mice model where a *SMAD3* knock‐out model presents with similar articular damages than in human osteoarthritis (Yang et al., 2001). Other joint diseases can also be found like osteochondritis dissecans that likely happened in one of our patients (patient 3, Data S2).

Osteoporosis has been reported in TGFβvs (Kirmani et al., 2010), as well as in MFS (Haine et al., 2015) and the importance of TGFβ pathway on the bone matrix metabolism is well known (Kirmani et al., 2010), the frequency of osteoporosis in SRD is not really known but could represent a third of patients (Schepers et al., 2018). Our data underline the high prevalence of decreased bone mass in SRD and more generally in TGFβvs (Kirmani et al., 2010) that can become an important issue in patients care.

Although various type of allergies, such as asthma, eczema and allergic conjunctivitis, have been previously described as frequent in SRD (Aubart et al., 2014) and are present in most of our patients (10/17, Table 1; Data S2), two of them (patient 10 and 17) made an anaphylactic shock (Data S2) which has not been described yet.

The systemic score of the revised Ghent Nosology (Loeys et al., 2010) shows the phenotypic overlap between SRD and MFS with 20% of our patients (4/20, Table 1) having positive score (⩾7). Nevertheless, this score is varying from 0 to 8, illustrating the high clinical variability of SRD and therefore the lack of power of such a score to detect *SMAD3* pathogenic variants as it was designed for MFS and does not include premature osteoarthritis which is more frequent and specific of SRD. Nevertheless, SRD are much less frequent than MFS and the use of multigene panels to diagnose connective tissue disorders with a vascular risk makes the development of a score dedicated to SRD not really helpful in clinical practice.

About 2/3 of patients have a missense variant (91/155) whereas 1/3 have a truncating variant (58/155) and less than 5% affects the splicing (6/155). Although no hot spot has been identified in *SMAD3* (Schepers et al., 2018), half of the *SMAD3* variants identified in our cohort are supposed to affect the MH2 domain (71/155 patients had a MH2 missense variant, Data S3).

From all these clinical and molecular data, we tried to establish genotype‐phenotype correlations, as it is relevant in clinical practice. For example, genotype‐phenotype correlations have been made, using a very large cohort of patients, to help the clinical management and the risk stratification in subjects with MFS (Faivre et al., 2007). Today, there are only few genotype‐phenotype correlations in TGFβ vasculopathies, made difficult owing to wide inter and intrafamilial phenotypic variability (Aubart et al., 2014; Hostetler et al., 2019; Laar et al., 2012) and often incomplete penetrance at pediatric age. Nevertheless, clinical and molecular characterization of novel patients is important to help the establishment of possible correlations and *in fine* to help clinical management.

In this work, we wanted to explore some aspects of the *SMAD3* phenotypic variability, asking the question of the involvement of specific variants with an “aortic tropism” in patients. Thus, we checked here if a correlation exists between the variant type (truncating vs. missense) and the occurrence of an AP, namely the presence of an aortic event (dissection, rupture or elective repair of the aorta) or aneurysm in the disease course. Moreover, to take in consideration a potential recruitment bias due to generally more severe symptoms in index cases, we also compared the phenotype of index cases only. Nevertheless, we did not find any correlation between the type of variants and the occurrence of an aortic disease in patients. This data are consistent with the recently published work by Hostetler et al. (2019) as they did not detect a statistical difference in the rate of aortic event in their cohort. Likewise, we did not detect any significant difference in the aortic disease occurrence between MH1 or MH2 missense variants and truncating variants, despite the fact that aortic events have been shown to happen earlier in life in patients with MH2 missense variants than in patients with truncating variants (Hostetler et al., 2019). Aortic dilatation is often asymptomatic, the age of apparition is then difficult to determine and not mentioned in the different reports. Furthermore, there is a bias between index cases (often diagnosed following an aortic event) and their relatives who benefit from a precocious vascular follow up and can therefore have an earlier diagnosis of aortic dilatation. Therefore, in our study, it was not possible to take the age in consideration in genotype‐phenotype correlations. Moreover, the lack of difference that we observe herein, could actually be due to a lack of statistical power. However, the p‐values that we obtained are very high and the analyzed sample (149 patients) is not so small, so if there is a difference, it is probably not major. Of note, Hostetler's study (Hostetler et al., 2019) recorded an AP solely for 177 of the 212 patients.

As some patients have vascular aneurysms or dissections in small arteries without AP, it would be interesting to investigate the vascular phenotype in SRD. The absence of a full vascular imaging in most of the patients reported in the literature makes the study of vascular phenotype difficult using these data. In addition, the phenotypic variability in the skeletal and joint features, in particular osteoarthritis, would also be interesting to investigate, as it appears as a frequent feature. However, establishment of genotype‐phenotype correlations based on these features in patients with *SMAD3* variant is made difficult given osteoarthritis and osteoporosis are common disorder in the general population, often banalized and underdiagnosed in the SRD patients, explaining the lack of data in the literature. Moreover, it appears from this work that aortic insufficiency and severe allergic phenomenon are probably also underestimated, unlike Charcot–Marie–Tooth‐like neuropathy, which was not found in our patients.

Thus, to date, there is no genotype‐phenotype correlation in the penetrance of the cardiovascular phenotype identified in the *SMAD3* gene. This is in line with the high variability observed in this syndrome, in particular the wide intrafamilial variability that is largely illustrated in this work. This also suggests that there are other factors impacting on the phenotype to be discovered and the presence of genetic modifiers can be one of them. Indeed, one of the family reported here (family 3) allows illustrating this phenomenon, with the probable effect of a variant in a genetic modifier influencing on the phenotypic expression and severity, as reported in MFS (Luyckx et al., 2019). Thus, the identification of genetic modifiers that could reveal, reduce or increase the *SMAD3*‐related phenotype seems to be an interesting and challenging area of research, especially as the use of NGS technology spreads out in routine genetic testing.

## CONFLICT OF INTEREST

None declared.
